# Report of the National Stock-grower’s Convention

**Published:** 1898-06

**Authors:** 


THE official report of the National Stock-growers' convention held at Denver, Col., in January last, has been received. It is its first report, but the vigor with which this movement was inaugurated is best told by the excellent reproduction of its proceedings in permanent form, and the scope of its work best understood by a perusal of the various aspects of its labors in the table of contents, including quarantine laws, railway transportation, tariff measures, diseases, laws of brands, foreign markets, etc.

